# Are External Cervical Orthoses Necessary after Anterior Cervical Discectomy and Fusion: A Review of the Literature

**DOI:** 10.7759/cureus.688

**Published:** 2016-07-14

**Authors:** Richard Camara, Olaide O Ajayi, Farbod Asgarzadie

**Affiliations:** 1 Department of Neurosurgery, Loma Linda University Medical Center; 2 Fontana Medical Center, Kaiser Permanente Southern California

**Keywords:** anterior cervical discectomy and fusion, external cervical orthoses, post-operative collar, cervical collar, cervical brace, neurosurgery, spine surgery

## Abstract

Introduction & Background: The use of external cervical orthosis (ECO) after anterior cervical discectomy and fusion (ACDF) varies from physician to physician due to an absence of clear guidelines. Our purpose is to evaluate and present evidence answering the question, “Does ECO after ACDF improve fusion rates?” through a literature review of current evidence for and against ECO after ACDF.

Review: A PubMed database search was conducted using specific ECO and ACDF related keywords. Our search yielded a total of 1,267 abstracts and seven relevant articles. In summary, one study provided low quality of evidence results supporting the conclusion that external bracing is not associated with improved fusion rates after ACDF.  The remaining six studies provide very low quality of evidence results; two studies concluded that external bracing after cervical procedures is not associated with improved fusion rates, one study concluded that external bracing after cervical procedures is associated with improved fusion rates, and the remaining three studies lacked sufficient evidence to draw an association between external bracing after ACDF and improved fusion rates.

Conclusion: We recommend against the routine use of ECO after ACDF due to a lack of improved fusion rates associated with external bracing after surgery.

## Introduction and background

Cervical collars have been used in patients pre and postoperatively for anterior cervical discectomy and fusion (ACDF) surgeries with the goal of cervical immobilization. Since studies have shown that cervical collars decrease cervical spine mobility, collar use has often been assumed to prevent further spinal cord injury [1-3]. Additional potential benefits of cervical collars include the restriction of neck flexion, extension, lateral tilt (bending), and rotation [1]. Other studies have shown that cervical immobilization reduces pain and provides spinal stability [2-3]. The benefits of cervical orthoses are not just physical but also mental since external collars also provide patients with an increased sense of security [4].

Common characteristics are shared by the variety of cervical orthoses that exist [1, 5-10]. To decrease cervical mobility, cervical orthoses are universally designed to provide an optimal fit against the jaw, occiput, and upper thorax [3]. While Halos are found to be more restrictive than soft collars [11], they do not completely eliminate mobility, despite the general consensus that rigid cervical orthoses are more limiting of cervical motion [2, 6, 8, 12-16]. Additionally, despite being less restrictive, soft collars may have an added benefit of increasing patient awareness due to enhanced proprioception [10, 17].

Some physicians recommend the use of postoperative cervical orthoses while others do not, and between surgeons who agree with postoperative collar usage the type of cervical orthoses and the duration of use are also topics of debate [18]. Because of this lack of professional consensus among spine surgeons regarding the use of external cervical orthoses (ECO) after ACDF, this review answers an important and relevant clinical question for spine surgeons performing ACDF procedures.

The purpose of this review is to evaluate the evidence for and against the use of ECO after ACDF. The question we ask is, “Does ECO after ACDF improve fusion rates?” By answering this question, we can improve patient outcomes, and if cervical orthoses are not recommended, reduce medical costs for patients.

## Review

This review adheres to the reporting recommendations established by Stroup et al., and the Meta-analysis of Observational Studies in Epidemiology (MOOSE) Group [19] and was conducted by doctors of medicine to ensure proper assessment of clinical studies. A PubMed database search was conducted using the following keywords: “cervical collar fusion,” “postoperative cervical immobilization,” “postoperative cervical collar,” “cervical immobilization fusion,” “postoperative cervical orthoses,” “cervical orthoses fusion,” “neck collar fusion,” “postoperative neck collar,” “postoperative surgical collar,” and “surgical collar fusion.” Our search yielded a total of 1,267 abstracts. These abstracts were individually reviewed, and full-text versions of relevant articles were obtained. Additionally, the related citations generated by PubMed and the bibliographies of relevant articles were reviewed. Studies investigating fusion rates after ACDF related to the presence or absence of external cervical collars were considered relevant. Studies that failed to meet this criterion were excluded. All studies obtained were in the English language, and no unpublished data were used. No search software or hand searching was used, and we did not establish contact with any authors of the reviewed papers. Due to the paucity of studies evaluating postoperative cervical spine stabilization after ACDF, we broadened our discussion to include a study evaluating complications after corpectomy/fusion (ACF). Finally, due to their direct relevance to the question under review, two questionnaires evaluating postoperative bracing practices among surgeons were included. All studies were critically appraised using the Grading of Recommendations Assessment, Development, and Evaluation (GRADE) approach developed by the GRADE working group and supported by The Cochrane Collaboration [20]. Because each study included for review chose to define fusion using slightly different criteria, rates of fusion success or failure reported in this review were calculated using the definitions established by each report.

In total, seven studies were included in this review. Using the GRADE approach, we ranked one of the studies as low quality of evidence and the remaining six articles as very low quality of evidence. A meta-analysis was not performed because the diversity of the studies included for review was too great. However, the total number of patients evaluated in five of the seven studies (two of the studies were questionnaires of surgeon preference) was 1,090. Table 1 summarizes each article included in this review.

**Table 1:** Table summarizing the included articles

Study	Campbell et al. 2009 [21]	Cauthen et al. 1998 [22]	Abbott et al. 2013 [23]	Jagannathan et al. 2008 [24]	Epstein 2007 [25]	Bible et al. 2009 [26]	Picket et al. 2004 [27]
Number of Patients	257	514	33	170	116	88	60
Study Design	Retrospective non-randomized analysis of braced vs non-braced groups after ACDF with anterior cervical plate.	Retrospective analysis of ACDF outcomes and outcome-relevant variables with a literature review (1975-1996) of non-instrumented ACFs	Randomized controlled trial comparing ACDF with and without external cervical orthoses (ECO)	Retrospective review of a prospective database investigating fusion rates and outcome measures after single-level non-instrumented ACDF without post-operative rigid cervical immobilization.	Prospective study evaluating the complications of single-level anterior corpectomy/fusion (ACF) using iliac crest autograft and dynamic ABC plates, with an average follow-up of 3.24 years (one year minimum).	Questionnaire recording the attitudes and preferences of spinal surgeons regarding post-operative bracing after specific spinal procedures.	Web-based survey of Canadian spine surgeons to determine current practices in management of patients undergoing ACDF
Quality of Evidence (Grade)	Low	Very Low	Very Low	Very Low	Very Low	Very Low	Very Low
Inclusion Criteria	Symptomatic single-level radiculopathy or myelopathy	Cloward’s ACDF procedure by the senior author for disc herniation or degeneration with intractable nerve or spinal cord compression from 1974 to 1994, with at least 2 years’ follow-up.	Age 18-65 years, ACDF procedure for nerve root compression refractory to conservative treatment >3 months; or diagnosis of cervical spondylosis, disc herniation, or degenerative disc disease.	Single level ACDF by the senior author for treatment of degenerative disease between June 1996 and June 2005.	Single-level ACF from 2000-2006 for contiguous 2-level pathology (disc disease, spondylosis, stenosis, and/or ossification of the PLL) with retrovertebral extension on magnetic resonance and computed tomography (CT) studies.	Spine surgeons in attendance at the “Disorders of the Spine” conference (January 2008, Whistler, Canada)	Canadian neurosurgeons and orthopedic spine surgeons with a clinical practice of >5% spine surgery.
Exclusion Criteria	Unclear post-operative bracing status.	Patients lost to follow-up, death incomplete medical records, cervical fractures or posttraumatic instability.	Lack of understanding of the Swedish language and previous ACDF procedure.	Traumatic or neoplastic disease, multilevel ACDFs, patients lost to follow-up.	None stated.	Questionnaire not returned, incomplete biographical information	No email response to invitation, declining to participate because spine surgery formed no or less than 5% of current practice.
Population	257 operative cases retrospectively divided into two groups 149 with external orthoses, 108 without external orthoses	514 records originally reviewed with only 348 patients analyzed (based on inclusion/exclusion criteria) for a total of 21 outcome and outcome-relevant variables, including cervical collar use.	33 patients undergoing ACDF randomly assigned into one of two groups: 17 with cervical collar and 16 without cervical collar	170 patients in a prospective database retrospectively evaluated for outcome relevant variables after ACDF.	116 patients undergoing single-level ACF were prospectively followed.	88 spine surgeons attending the “Disorders of the Spine” conference (January 2008, Whistler, Canada)	60 Canadian neurosurgeons or spinal orthopedic surgeons invited by email to complete a questionnaire.
Demographics	Groups were similar for age, gender, and Worker’s compensation; dissimilar for litigation, smoking, and working.	47% male, 53% female with an average age of 40 years. 202 (58%) one-level fusions; 129 (37%) two-level; 14 (4%) three level; 2 (0.6%) four-level; and 1 (0.3%) five-level. Graft source: allograft (70%) and autograft (30%)	The randomization process produced even group distribution for background characteristics of the patients and baseline variables.	73 (43%) female, 97 (57%) male, with a mean age of 53 years (median 56 years, range 34-67 years). 78 (46%) had only degenerative spondylosis, 55 (32%) had disk herniation, and 37 (21%) had radiographic evidence of both. 10 patients had history of previous single-level posterior cervical discectomies (6%) and 5 (3%) had prior multilevel cervical laminectomies with recurrent or residual symptoms. The operative level was at C3-4 in 28 patients (16%), at C4-5 in 29 (17%), at C5-6 in 71 (42%), and at C6-7 level in 42 (25%). 15 (9%) had undergone prior posterior cervical fusion.	52 females and 64 males with an average age of 45 (range 23-69). Average preoperative Nurick Grade was 3.19 (moderate spastic myelo-radiculopathy). 43 patients weighed over 200 lb, while 21 weighed over 240 lb.	Questionnaire distributed to 118 surgeons with 20 (25%) excluded. 55% of respondents were orthopedic surgeons and 45% were neurosurgeons. 66% affirmed completion of a spine fellowship. 60% were in private practice, and 40% were in academic practice. 24% had practiced for <5 years, 32% for 5-10 years, 27% for 10-15 years, and 17% for >15 years. 14% were currently practicing in countries other than the USA.	Email invitation was sent to 159 surgeons (59% neurosurgeons and 41% orthopedic surgeons). 72% were in academic positions. 18% had been in practice < 5 years, 27% from 6-10 years, 33% from 11-20 years, and 22% from 20-30 years. Spine surgery accounted for 54% of surgical practice for the responding neurosurgeons, and 70% of practice for the responding orthopedic surgeons.
Fusion Criteria	Defined as the presence of bridging trabecular bone, angulation of less than or equal 4° on flexion-extension radiographs, and absence of radio-lucencies.	Defined as radiographic absence of motion on flexion-extension lateral views. Fusion was recorded when bridging trabeculae were seen on radiographs, without motion or when perigraft lucency was seen without motion.	Defined as lack of qualitative motion of the interbody cage on post-operative flexion/extension radiographs.	Defined as lack of motion on postoperative dynamic images and trabecular bridging of the bone-graft interface on postoperative radiographs.	Included the documentation of bony trabeculation traversing the end plate-graft interface combined with the lack of lucency on 2D-CT. Also included the lack of translation, less than 1mm of motion between adjacent spinous processes, and less than 5 degrees of angulation.	n/a	n/a
Results	No significant differences in fusion success were seen between groups as assessed by independent radiologists. Higher rates of non-statistically significant fusion were reported in the non-braced group over all intervals. At 6 months, 89.8% fusion rate was reported in he braced group and 94.5% in the non-braced group achieved fusion (P = 0.379). At 24 months, 96.1% fusion was reported in the braced group and 100% in the non-braced group (P = 0.552).	No significant correlation was found between fusion and use of postoperative orthoses (86% fusion rate with cervical collar vs 81% fusion rate without collar).	Radiologists noted no qualitative difference in post-operative fusion rates or sagittal alignment between the cervical collar group and those not prescribed a post-operative collar. Radiographic fusion rates were 100% in both groups.	Postoperative radiographs demonstrated fusion in 160 patients (94%). The high fusion rate (94%) and overall favorable neurological outcomes (96%) associated with non-instrumented single-level ACDF with no postoperative collar indicates that this is an efficacious option in treating cervical spondylosis.	Initially, patients wore cervico-thoracic orthoses (CTO) until dynamic films and 2D-CT evaluation confirmed fusion, but since inadequate bracing was thought to have contributed to the delayed strut fractures in 7 (18.4%) of the 38 patients in the first 2 years of the study, the subsequent 78 patients undergoing surgery in the latter 4 years of the study used cervico-thoracic orthoses (CTO) for an additional 6 weeks (average 5.5 mo). No further delayed strut fractures were observed after this intervention.	Only a slight majority (56%) reported routine use of cervical or lumbar orthoses post-operatively. A common justification reported was that orthoses “slow down” patients and remind them to avoid certain activities which may compromise their clinical outcomes.	Surgeons recommended ECO for 92% of patients without anterior cervical plates and 61% of patients with anterior cervical plates for reasons including multilevel pathology, concern about bone strength or screw placement, patient discomfort, and the ‘routine.’
Study Limitations	We found no limitations in the ability of this retrospective study to compare fusion rates between braced and unbraced groups. Groups were dissimilar for smoking, but because the non-braced group had a higher percentage of smokers and a higher fusion rate, smoking as a confounding variable strengthens rather than weakens the conclusion that bracing does not improve fusion rates.	166 of 514 (32%) patient records were unavailable for follow-up. The number of braced and unbraced patients was also not specified	The study is substantially underpowered to detect differences in fusion rates between groups, as there are studies that report a non-fusion rate of approximately 2% when modern ACDF techniques are used (Marawar et al, 2010).	No intra-study comparison can be made between ACDF with external immobilization and ACDF without external immobilization since all patients were treated without rigid external immobilization.	The results/conclusions relevant to this literature review were made due to a change in protocol that occurred at study year 2 of 6. Additionally, changes in surgical technique made at year 2 of 6 could be a confounding variable. Also, this study investigated anterior ACF rather than ACDF. 43 patients weighed over 200 lbd, while 21 patients weighed over 240 lbs, limiting external validity.	The questionnaire required participants to assess their own practice patterns, subjecting their responses to recall bias. It is unclear whether this data truly reflects the opinions and preferences of the spine community at large, as a large proportion of the surgeons were fellowship trained (66%) and have academic affiliations (40%).	All surveys suffered from possible reporting bias, and a low response rate. The list of surgeons was compiled from membership information for the North American Spine Society, Canadian Spine Society, and the Canadian Congress of Neurological Sciences. The Canadian Orthopedic Association was excluded, based on the assumption that orthopedic spine surgeons would be captured by their membership in other organizations. This may have presented a disproportionate sampling of neurosurgeons
Conclusion	The use of cervical brace does not improve the fusion rate or the clinical outcomes of patients undergoing single level-anterior cervical fusion with plating and is probably unnecessary. The results of this study should be confirmed by randomized clinical trials of bracing versus no bracing or other similar studies of patients enrolled in current clinical trials.	Fusion rate is statistically unrelated to cervical collar use	The results of the study suggest that short-term cervical collar use post ACDF and interbody cage may help certain patients cope with initial post-operative pain and disability. Larger data collections are required to investigate health-related quality of life and fusion rates in patient with and without rigid collar use post ACDF surgery.	The results of the study suggest that use of post-operative cervical collar is unnecessary, as the immediate and long-term fusion rates did not appear to be affected by the lack of immobilization. A randomized controlled trial will be essential in determining the true benefit of external or internal fixation in patients who undergo single-level ACDF for cervical spondylosis.	The addition of 6 weeks of bracing to the clinical protocol eliminated delayed graft fractures.	While the most appropriate indications for postoperative bracing are yet to be elucidated, it is apparent that well designed clinical studies evaluating the relative efficacies of these diverse regimens are required so that evidence-based guidelines may be available to surgeons in the future.	Differences in technique persist not because they best address the variability of the disease process or variability among patients, but rather because there is variability among surgeons and their training.
Does ECO improve fusion rates after ACDF? (yes, no, unknown)	No	No	Unknown	No	yes	Unknown	Unknown

In the first study, Campbell et al., [21] performed a retrospective analysis of 257 patients divided into braced (149 patients) and non-braced (108 patients) groups without randomization after decompression and arthrodesis using allograft and anterior cervical plate. Although the data for this study were collected during a randomized control trial, the actual design of this study is retrospective. The rate of fusion at six months was not statistically different between braced (89.8%) and non-braced (94.5%) groups (p = 0.379). At 24 months, the rate of fusion was once again not statistically different between the groups, with 96.1% fusion in the braced group and 100% fusion in the non-braced group (p = 0.552). The results of this study indicate that external bracing after ACDF is not associated with improved fusion rates. We found minimal limitations in the ability of this study to retrospectively compare fusion rates between braced and non-braced groups that would suggest a high likelihood of bias. The population, intervention, control, and outcomes were all correctly designed to investigate the effect of external bracing on fusion rates. Using the ranking system developed by the GRADE working group, we rank the quality of this evidence as low (the highest possible for an observational study) due to the meeting of criteria for appropriate population, intervention, control, and outcomes [20].

In the second study, Cauthen et al., [22] performed a retrospective analysis of ACDF outcomes and outcome-relevant variables with a comprehensive literature review (1975-1996) of non-instrumented anterior cervical fusions. Three hundred forty-eight patients were analyzed for a variety of outcome-relevant variables, including cervical collar use. In this study, the fusion rates with and without a cervical collar were 86% and 81%, respectively. Unfortunately, only fusion percentages were provided; the actual numbers of braced and non-braced patients were not indicated. The fusion rates with and without cervical collar use were not statistically different, and the authors of this study concluded that fusion rates are unrelated to the use of orthoses. It should be noted, however, that 166 of 514 (32%) of the patient records were unavailable. This loss of patient data, although indicated as unavoidable by the study authors, severely limits the ability of the study to answer the question asked by this review. Using the criteria established by the GRADE working group, we rank the quality of this evidence as very low due to the loss of follow-up even though the study met criteria for appropriate population, intervention, controls, and outcomes [20].

In the third study, Abbott et al., [23] conducted a randomized controlled trial with 33 patients ACDF without ECO (16 patients) to ACDF with ECO (17 patients). Although the rate of fusion in both groups was 100%, the effect of bracing on fusion rates cannot be determined due to low patient numbers. Even though the design of this study is a randomized controlled trial, the quality of evidence is lower than expected. Being a pilot study, the population size was too small to properly evaluate the effect of external bracing on fusion rates. Additionally, patients and investigators were not blinded to postoperative treatment allocation. Even though this study met criteria established by the GRADE working group for appropriate intervention, controls, and outcomes, we rank the quality of this evidence as very low due to the low population size and the lack of blinding [20].

In the fourth study, Jagannathan et al., [24] conducted a retrospective review of a prospective database investigating fusion rates and other neurological outcome measures after ACDF without the use of intraoperative plate placement or the use of postoperative rigid cervical immobilization in 170 patients. Fusion was recorded in 160 of the 170 (94%) patients. Although 94% is an excellent fusion rate, the ability of this study to answer the question asked by this review is limited, since fusion rates were only investigated in patients without external bracing. The authors concluded that high fusion rates without external bracing render such orthoses as unnecessary. Due to meeting the criteria established by the GRADE working group for appropriate population, control, and outcomes but failing to meet criteria for appropriate intervention, lack of a braced group, we rank the quality of this evidence as very low [20].

In the fifth study, Epstein [25] performed a prospective study evaluating complications of single-level anterior corpectomy/fusion (ACF) using iliac crest autograft and dynamic ABC plates (Aesculap, Tuttlingen, Germany). Initially, all patients were braced using cervicothoracic orthoses (CTO) until dynamic films and 2D-CT evaluation confirmed fusion. However, in the first two years of the study, 7 of 38 (18.4%) patients experienced delayed strut fractures. Thus, CTO use for the remaining 78 patients operated on during years 4-6 of the study was extended for an additional six weeks past the point of radiographic fusion confirmation. After this change in protocol, no further delayed strut fractures were observed. The authors posit that reduction of delayed strut fractures was associated with the extended CTO use. The ability of this study to answer the question asked by this review is limited because the intervention was single-level ACF rather than ACDF. Additionally, the recommendation to extend the time of external bracing was made based on a 'before and after' study design change that occurred at year 2 of 6. Furthermore, changes in surgical technique at year 2 make it impossible to ascertain whether the improved outcomes during years 2 through 6 were due to extended external bracing time. In addition, the weight of the patients included in this study may be a confounding variable with 43 of the 116 patients weighing over 200 lbs., and 21 of the patients weighing over 240 lbs. Even though this study met criteria established by the GRADE working group for appropriate outcomes, we rank the quality of this evidence as very low due to several limitations, including a non-ACDF procedure, a 'before and after' study design, and confounding variables, i.e., obesity and surgical procedure changes [20].

In the sixth study, Bible et al., [26] prepared a questionnaire to record the attitudes and preferences of spine surgeons regarding postoperative bracing after specific spinal procedures. One hundred eighteen questionnaires were distributed to spine surgeons attending the “Disorders of the Spine” conference hosted in Whistler, Canada in January 2008. Eighty-eight questionnaires were included in the analysis. Results indicated that 56% percent of surgeons routinely use some type of external orthoses to complement the surgical treatment of the cervical and lumbar spine. Surgeons most commonly used external bracing to reduce motility while maintaining a safe level of activity. Because the lowest rank established by the GRADE working group for quality of evidence is very low and due to the natural tendency of all questionnaires to have a high bias and subjectivity, no recommendations for clinical practice can be made based upon the results of this study, and we rank the quality of this evidence as very low [20]. Although, this study is a poor source of information for clinical decision making, the fact that 56% of the surgeons routinely used external bracing while 44% did not make a clear argument for the necessity of this review.

In the seventh and final study included in this review, Pickett et al., [27] conducted a web-based survey of Canadian spine surgeons to determine current practices in the management of patients undergoing ACDF. Invitations to participate in the questionnaire were sent to 159 Canadian neurosurgeons or spinal orthopedic surgeons. Sixty surgeons were included in this analysis. According to the survey, surgeons recommended ECO for 92% of patients without anterior cervical plates and 61% of patients with anterior cervical plates. Surgeons indicated “multilevel pathology, concern regarding bone strength or screw placement, patient discomfort, and the ‘routine’” as reasons for the use of external bracing. Using criteria established by the GRADE working group, we rank the quality of this evidence as very low [20]. Because no clinical recommendation can be made based upon the results of this study since questionnaires have no intervention or control and a tendency for bias. Although, once again, the inconsistent recommendation of external orthoses among spine surgeons argues for the necessity of this review.

In summary, one study provided low quality of evidence results supporting the conclusion that external bracing is not associated with improved fusion rates after ACDF [21]. The remaining six studies provided very low quality of evidence results. Two of these studies concluded that external bracing after cervical procedures is not associated with improved fusion rates [22, 24]. One of these studies concluded that external bracing after cervical procedures is associated with improved fusion rates [25]. The remaining three studies lacked sufficient evidence to draw an association between external bracing after ACDF and improved fusion rates [23, 26-27].

On a side note, patient compliance is one important topic we felt should have been addressed by these studies but was not. Unfortunately, none of these studies addressed rates of postoperative collar compliance [21-27]. We cannot exclude the possibility that low patient compliance is a confounding variable in the studies showing no difference between external orthoses and no external orthoses. If patients with low compliance for external orthoses had been excluded from the external orthoses groups in these studies, a statistical difference in fusion rates may have been observed. We conclude that although this lack of information is unfortunate, patient compliance is a reality of practicing clinical medicine, and clinical decision-making should take into account poor patient compliance. Therefore, although these studies do not address patient compliance, they still effectively answer the question under review through an intention-to-treat study design.

Additionally, length of collar usage differed among studies. To simplify the analysis of our proposed question, we treated collar wearing in a binary nature, worn or not worn, but in reality some patients were instructed to wear ECO until six weeks after radiographic fusion was observed (possibly 12 or more weeks) [25] while other patients were instructed to wear ECO for as little as one week [27]. Some surgeons even scaled the size of the recommended bracing time period with the number of spinal segments operated on [26]. Furthermore, wearing instructions differed among studies. Some patients were instructed to continuously wear ECO [25] while other patients were instructed to wear ECO only during the day or when moving around [23].

Finally, the use of cervical orthoses after ACDF is not without complications. Even though some of these sound extreme, reported complications of ECO include skin breakdown and damage [1, 3, 28], difficulty swallowing, coughing, difficulty breathing, and vomiting [29-30]. Other complications include marginal mandibular nerve palsy with long-term sensory compromise [31], potential increase in intracranial pressure [32], possible delayed extubation or difficulty weaning from the ventilator [29], potential exposure to the transmission of blood-born diseases [8], pressure points at sacrum, heels, and elbows secondary to general immobility [29, 33], decubitus ulcers [1, 34], and skin necrosis [35].

## Conclusions

Based on the highest level of evidence and until a higher quality of evidence is available, we recommend against the routine use of ECO after ACDF due to a lack of improved fusion rates associated with external bracing after surgery. External bracing should be used only in patients with a specific need unrelated to fusion improvement in which the benefits of external bracing outweigh the risk of collar-related complications. Currently, the highest level of evidence supporting this conclusion is a retrospective study in which we found minimal design limitations [21]. The results of two additional studies also support this conclusion; however, we found limitations in their design [22, 24]. It should be noted that these studies did not report patient compliance. Thus, the effects of bracing on fusion rates in these studies may have been affected by the patients' compliance with bracing. In addition, the potential benefits of bracing may have been masked by other aspects of bracing, such as limited range of motion and reduced activity. Finally, some of these studies used differing techniques and hardware, which made comparison difficult. Further studies focusing on patient outcomes are necessary to further clarify ECO guidelines, including randomized studies of ACDF with or without ECO.
